# Tonotopic Ca^2+^ dynamics and sound processing in auditory interneurons of the bush-cricket *Mecopoda elongata*

**DOI:** 10.1007/s00359-023-01638-6

**Published:** 2023-05-24

**Authors:** T. Bayley, B. Hedwig

**Affiliations:** Department of Zoology, Cambridge, CB22 3EJ UK

**Keywords:** Optical imaging, Oregon green, Intracellular loading, Frequency processing, Contralateral inhibition

## Abstract

Two auditory neurons, TN-1 and ON-1, in the bush-cricket, *Mecopoda elongata*, have large dendritic arborisations which receive excitatory synaptic inputs from tonotopically organised axonal terminals of auditory afferents in the prothoracic ganglion. By combining intracellular microelectrode recording with calcium imaging we demonstrate that the dendrites of both neurons show a clear Ca^2+^ signal in response to broad-frequency species-specific chirps. Due to the organisation of the afferents frequency specific auditory activation should lead to local Ca^2+^ increases in their dendrites. In response to 20 ms sound pulses the dendrites of both neurons showed tonotopically organised Ca^2+^ increases. In ON-1 we found no evidence for a tonotopic organisation of the Ca^2+^ signal related to axonal spike activity or for a Ca^2+^ response related to contralateral inhibition. The tonotopic organisation of the afferents may facilitate frequency-specific adaptation in these auditory neurons through localised Ca^2+^ increases in their dendrites. By combining 10 and 40 kHz test pulses and adaptation series, we provide evidence for frequency-specific adaptation in TN-1 and ON-1. By reversible deactivating of the auditory afferents and removing contralateral inhibition, we show that in ON-1 spike activity and Ca^2+^ responses increased but frequency-specific adaptation was not evident.

**Supplementary Information:**

The online version contains supplementary material available at 10.1007/s00359-023-01638-6.

## Introduction

To understand the role of a single neuron within a network, the location of its synaptic inputs and the processing within its dendrites need to be studied (review: London and Häusser 2005). Topographic maps, which are found ubiquitously in the nervous systems of mammals (Thivierge and Marcus 2007; Derdikman and Moser 2010) and insects (Murphey et al. 1980; Peron et al. 2009; Hildebrandt 2014), can facilitate efficient processing of synaptic inputs to a neuron’s dendrites. If a neuron receives synaptic inputs from different regions of a sensory map on separate dendritic branches, this allows for localised post-synaptic dendritic processing (Koch and Segev 2000; London and Häusser 2005; Peron et al. 2009). Topographic maps occur in brain regions which respond to auditory stimulation, where neurons are commonly arranged into maps of frequency, known as tonotopic maps (e.g., mice: Issa et al. 2014; gerbils: Budinger et al. 2013; humans: Saenz and Langers 2014). Tonotopic maps also occur in the auditory neuropiles of insects (review: Hildebrandt 2014; Hedwig and Stumpner 2016). In bush-crickets, auditory afferents from the ears on the forelegs project to an auditory neuropile in the prothoracic ganglion and their axons are arranged such that low-to-high frequency sound is represented in an anterior-to-posterior direction (Oldfield 1983; Römer 1983; Stölting and Stumpner 1998; Baden and Hedwig 2010). Several interneurons responding to auditory stimulation have large dendritic arborisations (extending over ~ 300 μm), which overlap with this neuropile (Römer et al. 1988; Stumpner and Molina 2006). It is therefore likely that these large interneurons receive synaptic input from auditory afferents tuned to different frequencies in spatially separate dendrites, arranged according to the tonotopic organisation of the auditory afferents. Previous work has used imaging techniques to identify sound-induced Ca^2+^ increases in two auditory neurons in Orthoptera: an inter-segmental auditory neuron, TN-1, in the bush-cricket, *Neoconocephalus triops* (Triblehorn and Schul 2013; Presern et al. 2015) and a local auditory neuron, ON-1, in the cricket (Sobel and Tank 1994; Baden and Hedwig 2007). In both neurons, Ca^2+^ has been implicated to cause a reduction in neuronal excitability through the activation of Ca^2+^ -sensitive K^+^ channels. These channels are associated with activity-dependent spike-rate reduction in a wide range of animals (reviews: Faber and Sah 2003; Stocker 2004), including both invertebrates (Sobel and Tank 1994; Peron and Gabbiani 2009; Wessel et al. 1999), and vertebrates (Sanchez-Vives et al. 2000; Savić et al. 2001). If the activation of auditory afferents induces a Ca^2+^ increase in the dendrites of auditory interneurons at the position of synaptic input, this increase should be organised according to the tonotopic arrangement of the afferents. If Ca^2+^ activates K^+^ channels, this would result in a reduced response only in dendrites receiving input from afferents tuned to a particular frequency range, and so should allow for frequency-specific dendritic adaptation at the level of the interneuron (Presern et al. 2015), in addition to adaptation at the level of the afferent population. Previous studies in the bush-cricket, *N. retusus*, have shown that the large auditory interneuron TN-1 adapts to the conspecific call, a rapidly repeated series of pulses in the range of 15 kHz, but remains able to respond to the 30–50 kHz calls of predatory bats (Schul et al. 2012). In a separate context, bush-crickets often inhabit areas with high levels of background noise, such as rainforests, and several interneurons have been shown to adapt to the background noise whilst remaining able to respond to the signals of conspecifics (Schmidt and Römer 2011; Hartbauer et al. 2012a). Frequency-specific adaptation has also been observed in other species including the barn owl (Gutfreund and Knudsen 2005) and rat (Malmierca et al. 2009) and is often used to study the general principles of stimulus-specific adaptation and novelty detection, which are widespread in sensory processing (Nelken and Ulanovsky 2007; Nelken 2014). As a step towards understanding dendritic processing in the auditory system of the bush-cricket, *Mecopoda elongata*, which use a broad band communication signal (Siegert et al. 2013), we analysed two interneurons, TN-1 and ON-1, which have extensive dendritic arborisations in the auditory neuropile of the prothoracic ganglion. By iontophoretic injection of the calcium indicator, Oregon Green BAPTA-1, the spatio-temporal patterns of Ca^2+^ increases have been characterised in response to species-specific chirps and pure tone stimulation. Intracellular recordings allowed a comparison between the Ca^2+^ signals and the spiking activity of each neuron.

## Materials and methods

### Experimental animals

Bush-crickets (*Mecopoda elongata*) were taken from a colony in the Department of Zoology, University of Cambridge, and originally derived from Malaysia. Animals were kept at 21–23 °C on a 12:12 h light:dark cycle and fed with iceberg lettuce, muesli and fish flakes.

### Dissection

**Fig. 1:**
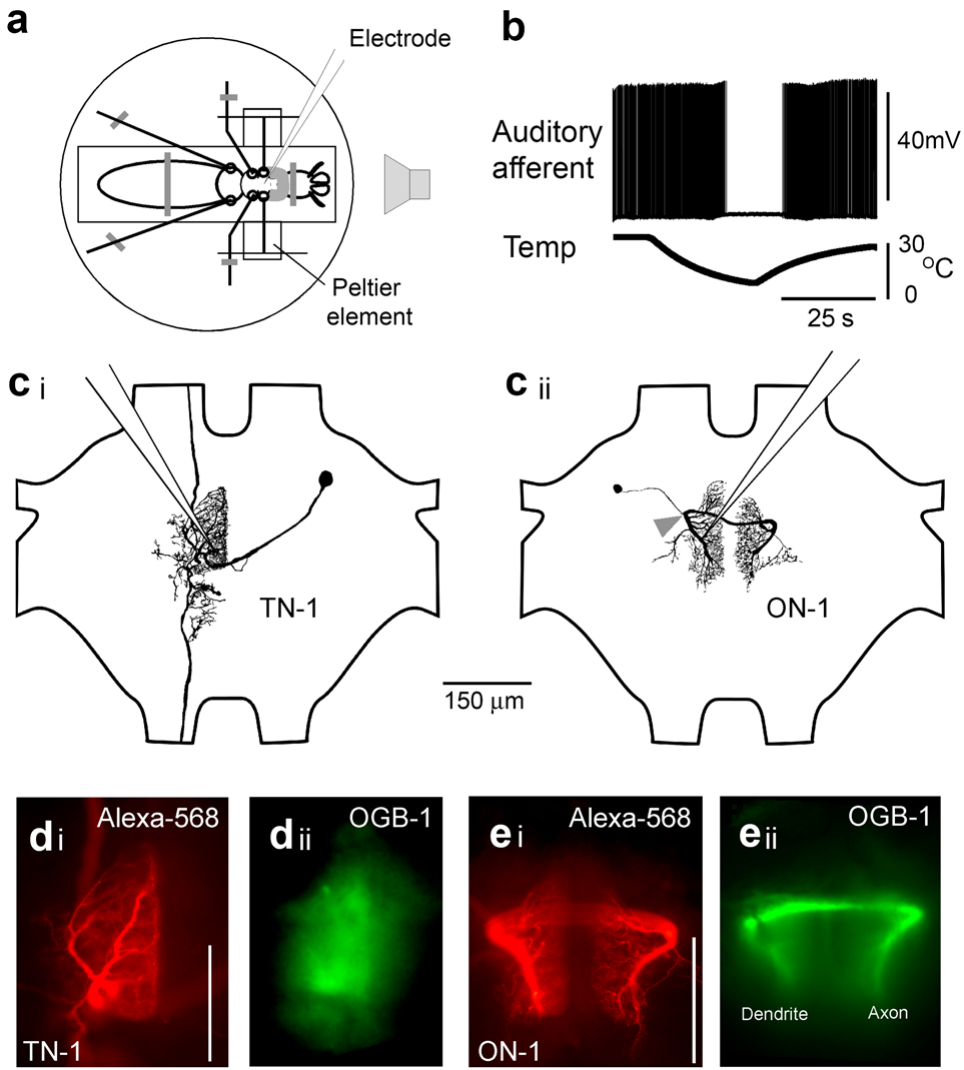
**a** Experimental setup for intracellular recording and optical imaging in the bush-cricket *Mecopoda elongata.* The prothoracic ganglion was exposed to record from TN-1 or ON-1. Peltier elements attached to the tibiae allowed reversible cooling of the auditory afferents. **b** Intracellular recording from an auditory afferent during cooling and repetitive stimulation with 70 dB SPL, 10 kHz tones (not shown), cessation of spiking occurred at 7–10 °C. **c**i, ii Morphology of TN-1 and ON-1 after staining with Alexa-568 and clearing. Position of electrodes in the large dendrites indicated. Arrowhead points to putative position of spike-generating zone (SGZ) in ON-1. **d**i and **e**i Morphology of TN-1 and ON-1 revealed after fixation and clearing, image taken with wide-field microscopy. **d**ii and **e**ii In vivo Ca^2+^ signal as indicated by intracellular OGB-1 staining, averaged from 50 frames of imaging camera with dendrites and axon of ON-1 indicated. Scale bars: 100 μm

Bush-crickets were restrained ventral-side up in a small non-conducting plastic dish containing Plasticine (Fig. 1a), this was necessary to isolate the animals electrically from Peltier elements used to cool the forelegs. The ventral prothoracic cuticle was removed before spreading the coxae of the forelegs to provide sufficient space for a water-immersion objective for imaging. Melted wax was used to build a ring around the opening, avoiding excessive heat which would damage the auditory afferents. The ganglion was covered with saline which had mM ionic concentrations of: NaCl–35, KCl–10, CaCl2–7, NaHCO3–8, MgCl2–1, TES–4.8, trehalose–4.4. Tracheae and fatty tissue were removed to expose the prothoracic ganglion. All prothoracic nerves and connectives were cut, except for nerve five containing the auditory afferents; the meso- and metathoracic ganglia were also removed. Although severing prothoracic nerves and connectives may have an impact on the response properties of the neurons studied, it was necessary as the mechanical stability of the prothoracic ganglion was essential for imaging. A stainless-steel platform was slid under the prothoracic ganglion, and a metal ring was sometimes pushed gently on top to improve stability.

### Reversible deactivation of auditory afferents

In crickets ON-1 receives inhibition from its contralateral partner neuron (Selverston et al. 1985). To analyse the effect of contralateral inhibition in the *M. elongata* ON-1, the auditory afferents within each foreleg were reversibly deactivated by cooling (Baden and Hedwig 2010; Zhang and Hedwig 2019). The tibia was held 90° from the horizontal with the tarsi waxed to a thin wire and the femur of each foreleg was secured against a 10 × 10 mm Peltier element (Peltron Technik, Fürth, Germany) using Plasticine. Temperatures of 5–10 °C were sufficient for total and reversible cold deactivation of auditory afferents (Fig. 1b).

### Sound stimulation

Sound patterns were generated in Cool Edit Pro 2.0 (Syntrillium now, Adobe Audition Systems, CA, USA). A Monitor 02 US sound card was used (Musiland Audio Labs, Beijing, China), capable of 24-bit 192 kHz sample rate playback. Sounds were presented from a single NEO 13 s speaker (Sinuslive, Conrad Electronik, Kaltenkirchen, Germany) placed directly in front of the animal, 10 cm from both ears. Sound was calibrated to ± 1 dB SPL with a 4939 microphone and 2610 amplifier (Bruel & Kjaer UK Ltd., Royston, UK). To test for harmonic distortion, the sound signal was recorded with a Powerlab 8/30 data acquisition system (AD Instruments, Oxford, UK) at 100 kHz and analysed in Cool Edit Pro. No distortion was discernible above background noise for any frequency used. For playback, natural species-specific chirps (Siegert et al. 2013) were presented at 70 dB SPL. To test for tonotopic Ca^2+^ signals during imaging, sound pulses were presented of 5–20 kHz in 5 kHz steps, or 10–40 kHz frequency in 10 kHz steps; three of four recordings of ON-1 were tested with 5–20 kHz pulses, and one with 10–40 kHz pulses, where all three recordings of TN-1 were tested with 10–40 kHz pulses. Short, 20 ms sound pulses were used to elicit Ca^2+^ responses. Pulses were presented at 6 s intervals. To test for frequency-specific adaptation during intracellular recordings, a series of 10 ms pulses was first presented, repeated at 20 ms intervals. After 2.5 s, a second series of pulses was presented in the silent intervals between the pulses of the first series, repeated at 200 ms intervals. The frequency of the pulses of the first and second series was varied, details given in the text.

### Electrophysiology

Microelectrodes were made from borosilicate glass capillaries (Hilgenberg GmbH, Malsfeld, Germany) of 1 mm outer diameter and 0.7 mm inner diameter, pulled to 40–60 MΩ resistance using a Zeitz DMZ Universal Puller (Zeitz Instruments GmbH, Martinsried, Germany). These electrodes allowed for better passage of dye, as standard thick-walled electrodes tended to become blocked when used with calcium indicators. For imaging experiments, electrode tips were back-filled with a mixture of 450 µM Oregon Green BAPTA-1 (OGB-1, Molecular Probes, Life Technologies Ltd. Paisley, UK) and 0.1% Alexa Fluor®-568 (Alexa-568, Molecular Probes), with 1 M KAc (Sigma-Aldrich, Gillingham, Dorset) in the shaft. Dyes were dissolved in UltraPure™ water (Invitrogen, Life Technologies Ltd. Paisley, UK), to ensure a low Ca^2+^ concentration and to avoid crystallisation of OGB-1. To obtain low-noise intracellular recordings, electrodes were filled only with 1 M KAc. The prothoracic ganglion was viewed using a Leica DM-LFS microscope (Leica Microsystems, Wetzlar Germany), with the electrode positioned with a Leitz micromanipulator (Leica Microsystems). To soften the ganglion sheath, a few crystals of protease (Type XIV, Sigma-Aldrich) were dissolved in the saline surrounding the ganglion for 15 s. The ganglion was then thoroughly rinsed with saline to avoid damage to tissue. Neurons were identified from their response properties and recorded in their dendrites (Fig. 1c). The electrode signal was amplified with a SEC-10x amplifier (npi electronic GmbH, Tamm, Germany) and sampled at 50 kHz for analysis offline in Spike2 software (Cambridge Electronic Design, Cambridge, UK). Depth measurements of the electrode tip position were made using a Mitutoyo 543–251B gauge (Mitutoyo Ltd., Andover, UK). Dyes were introduced with 3–5 nA hyperpolarising current for at least 15 min. Imaging was conducted 30–60 min after filling to allow the dye to spread. To view the neurons after dye-filling and imaging (Fig. 1d, e), the ganglion was removed and fixed in 5% paraformaldehyde, then dehydrated in a series of 70, 90, 95 and 100% ethanol for 30 min each, and cleared in methyl-salicylate (Sigma-Aldrich).

### Optical imaging

Experiments used a DM-LFS microscope (Leica Microsystems, Wetzlar, Germany) equipped with long distance 4× and 10× air objectives and 10× and 20× water-immersion objectives. The focal plane was adjusted to achieve maximum acuity for the dendrites of interest. An Optoscan monochromator (Cairn Research Ltd., Kent, UK) was used to emit light of 488 ± 15 nm (for OGB-1) or 568 ± 15 nm (for Alexa-568), uniformly onto the sample. Light passed through either a GFP (525 ± 50 nm range, Leica Microsystems), or Texas Red (630 ± 75 nm range, Chroma Technology, Vermont, USA) emission filter, before being captured by an iXon DV887DCS-BV cooled CCD camera (Andor Technology Ltd., Belfast, UK) at frame rates of 20 or 50 Hz. Pixels were averaged 2x or 4x across the x and y plane, providing 256 × 256 or 128 × 128 pixel resolution, respectively. For synchronisation, trigger pulses drove the capture mode of the camera and were recorded simultaneously with electrophysiological data. Images were acquired using iQ 2 software (Andor). Images were further analysed in ImageJ (National Institutes of Health, Bethesda, MD, USA) and Spike2. Images were stabilised for deviations in the x and y dimensions in ImageJ. The outline of the dendrites could clearly be seen and guided hand-drawn regions of interest (ROIs). From these, fluorescence values were extracted and are expressed as the relative percentage change in fluorescence from baseline fluorescence without stimulation, ΔF/F (%). The mid-point of fluorescence in the antero-posterior axis of the neurons was calculated for a subset of experiments based on calculations for the centre of mass of an object. For a rectangular ROI, the mean relative fluorescence change for all rows of pixels parallel to the medio-lateral axis was calculated. This mean relative fluorescence change was used as a weighting factor. Multiplication of the distance from the anterior-most position by the weighting factor, and division by the sum of all weighting factors, provided the mid-point of fluorescence change in the antero-posterior axis (details: Bayley and Hedwig 2018).

### Data analysis

Data were processed in Spike2 (Cambridge Electronic Design, Cambridge, UK), ImageJ and Excel (Microsoft Corporation, Redmond, WA, USA). Statistical tests were carried out in R (R Foundation, Vienna, Austria). Data were tested with a Shapiro–Wilk test, due to the small sample size confirmation of normality may require cautious interpretation. Paired and unpaired two-tailed two-sample t-tests were used, and data are presented as mean ± standard error, unless otherwise stated.

## Results

### Time course of Ca^2+^ responses

Based on previous imaging experiments (Baden and Hedwig 2007, 2010), we expected auditory evoked Ca^2+^ signals in the two auditory interneurons, TN-1 and ON-1. To characterise the response of each neuron, a large dendrite was intracellularly impaled with a microelectrode (Fig. 1c), and the calcium indicator OGB-1 was introduced to monitor intracellular Ca^2+^ dynamics (Fig. 1d, e). The position of the putative spike-generating zone (SGZ) in ON-1 was estimated relative to the dendrites and primary neurite (arrowhead in Fig. 1c ii; cf. Baden and Hedwig 2007).

**Fig. 2:**
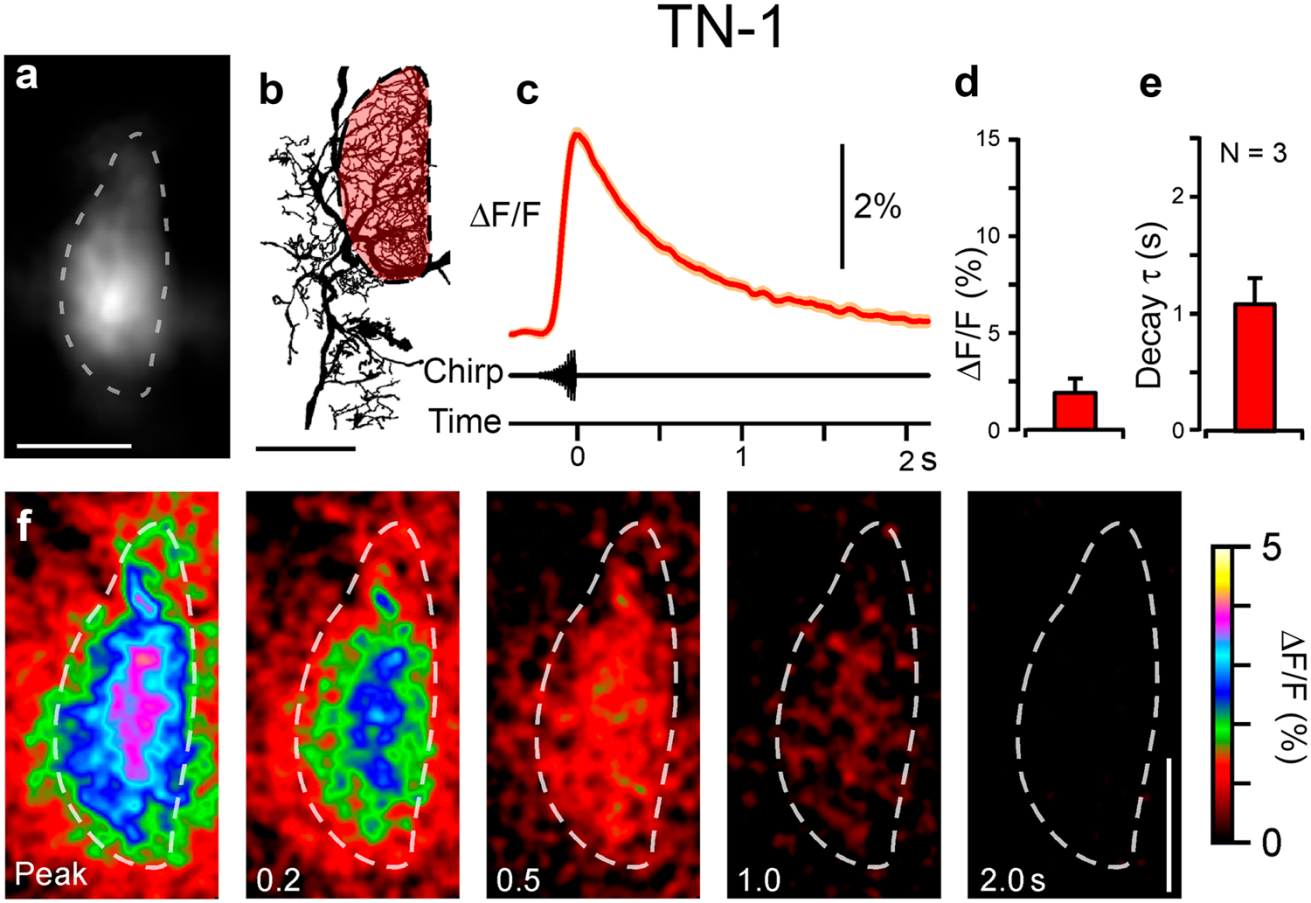
Ca^2+^ response in TN-1 to presentation of a chirp. **a** Baseline fluorescence without stimulation of OGB-1 in a live preparation, averaged from 100 frames before stimulus presentation, ROI drawn around dendrites. **b** Drawing of TN-1 from Alexa-568 staining in the same animal after fixation and clearing. Dendritic region highlighted in red corresponds to ROI in (**a**). **c** Mean ΔF/F from 12 presentations of a chirp in one animal. **d** Mean ΔF/F, and **e** time constant of decay for Ca^2+^ signal after presentation of a chirp (N = 3). Axes scaled for comparison with ON-1 (Fig. 3). **f** Snapshots of fluorescence on presentation of a chirp, mean of 12 presentations in one animal. Numbers refer to time from peak Ca^2+^ fluorescence in seconds. Dashed line corresponds to ROI in (**a**). Scale bars: 100 μm

We first tested Ca^2+^ induced fluorescence response to a species-specific chirp of 280 ms duration. The exact timing of the stimulus onset could not be measured, as each chirp commences with a gradual increase in amplitude (Fig. 2c). In both neurons, Ca^2+^ increased with the gradual onset of a chirp, reaching its maximum within 40–60 ms of the end of a chirp, or 2–3 frames of imaging. In the dendritic field of TN-1 (Fig. 2a, b), playback of a chirp induced a peak ΔF/F of 1.9 ± 0.8%, occurring within 330 ± 10 ms (Fig. 2c, d; Movie 1; N = 3, 10–20 presentations per experiment). The decay of fluorescence was uniform across the dendritic regions of the cell, with a time-constant τ_d_ of 1.1 ± 0.2 s (Fig. 2e, f).

**Fig. 3:**
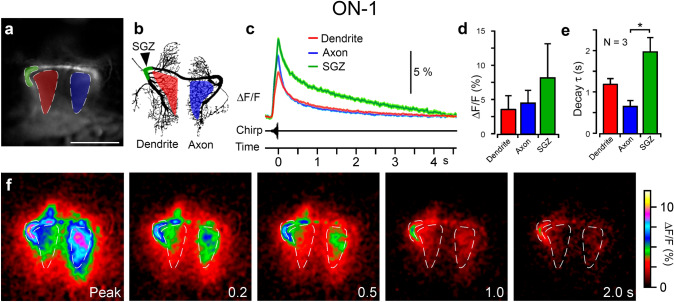
Ca^2+^ response in ON-1 to presentation of a chirp. **a** Baseline fluorescence without stimulation in live preparation, averaged from 100 frames before stimulus presentation, with ROIs indicated around dendrites (red), axonal neurites (blue) and putative SGZ (green). **b** Drawing of ON-1 from Alexa-568 staining after fixation and clearing, with highlighted areas corresponding to ROIs in (**a**). **c** Mean ΔF/F from 19 presentations of a chirp in one animal. **d** Mean ΔF/F and **e** time constant of decay for presentation of a chirp (N = 3), for dendrite (red), axon (blue) and putative SGZ (green). **f** Time-course of fluorescence decay on presentation of a chirp, mean of 19 presentations in one animal. Numbers refer to time from peak Ca^2+^ fluorescence signal. Dashed lines correspond to ROIs in (**a**). Scale bar: 100 μm

In the local neuron ON-1 the response of different compartments could be compared (Fig. 3a, b). The fluorescence increase to a chirp was larger than in TN-1, and differed in amplitude throughout the neuron; the axon gave 4.5 ± 1.9% ΔF/F, occurring within 330 ± 20 ms; the dendrite gave 3.6 ± 2.0% ΔF/F, occurring within 310 ± 30 ms; and the putative spike-generating zone (SGZ) gave 8.2 ± 4.9% ΔF/F, occurring within 320 ± 30 ms (Fig. 3c, d, f; Movie 2; N = 3, 10–20 presentations per animal). Relative to the dendritic region, the axon showed 1.4 ± 0.3 times greater ΔF/F (*p* = 0.031 dendrite, paired t-test), whereas the SGZ showed 1.8 ± 0.3 times greater ΔF/F (*p* = 0.027 dendrite; *p* = 0.61 axon). Neither of these were significantly different from each other after Bonferroni correction (*p* = 0.025). The rate of decay of fluorescence also varied between the axon with τ_d_ of 0.7 ± 0.1 s; the dendrites with τ_d_ of 1.2 ± 0.1 s; and the SGZ with τ_d_ of 1.9 ± 0.4 s (Fig. 3c, e, f). Of these, τ_d_ of the SGZ differed significantly from the dendrite (*p* = 0.03; SGZ dendrite, *p* = 0.30; axon dendrite, *p* = 0.55, ANOVA, Tukey post-hoc). Overall, in both neurons, a significant rapid Ca^2+^ increase occurred in response to sound, which decayed more slowly.

### Tonotopy in the Ca^2+^ response of TN-1

**Fig. 4:**
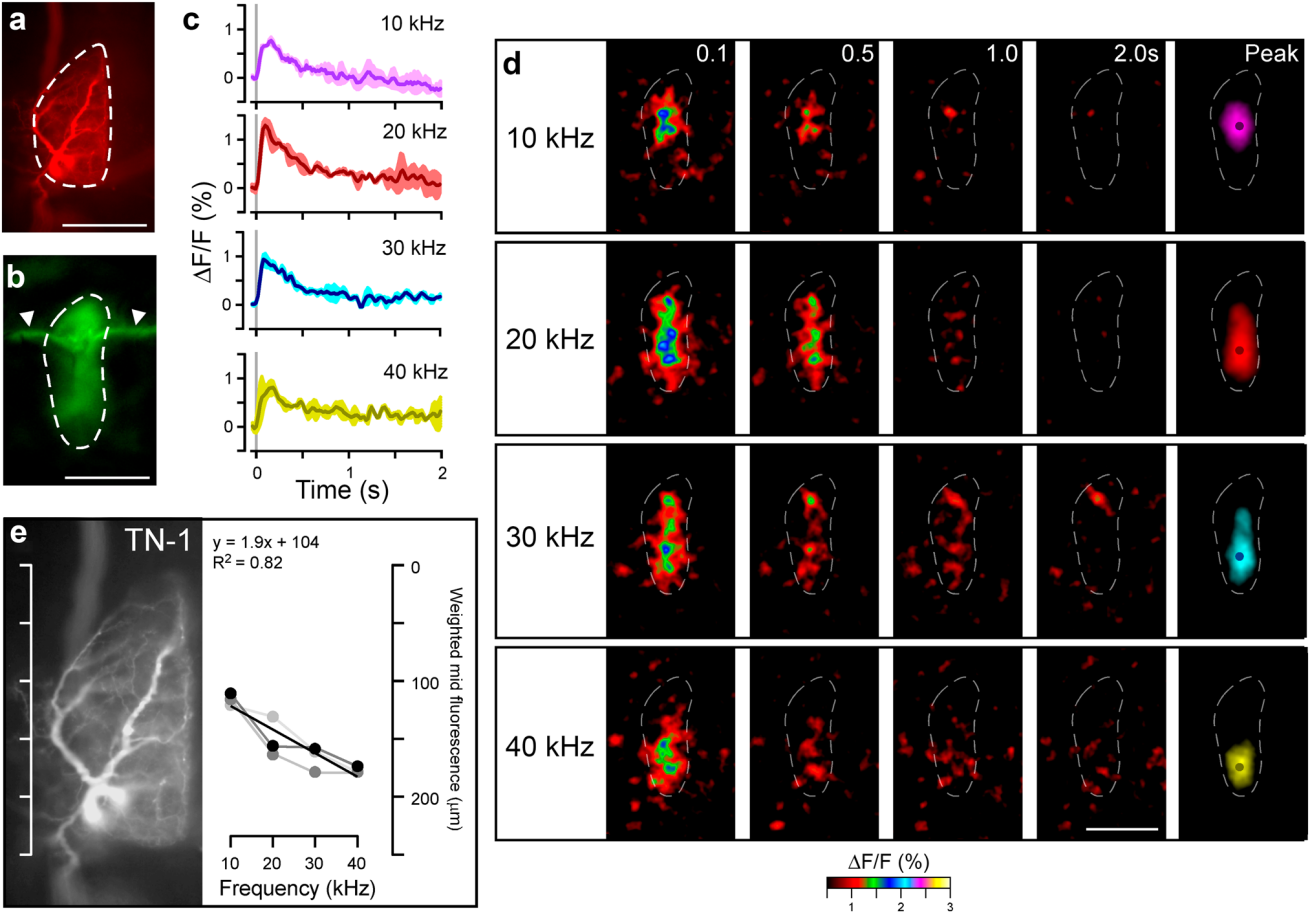
Ca^2+^ response of TN-1 to pure-tone sound pulses. **a** Image of Alexa-568 staining after fixation and clearing, with ROI indicated around dendrite. **b** Baseline fluorescence of OGB-1 without stimulation in live preparation averaged from 100 frames prior to stimulation. Arrowheads indicate marks on ganglion surface which are not part of the neuron. **c** Mean ΔF/F in single animal from ROI indicated in (**b**) after presentation of 20 ms, 70 dB SPL sound pulses of 10, 20, 30 and 40 kHz frequency indicated by grey bars, 3–5 presentations per frequency. **d** Mean ΔF/F from 3–5 presentations of 20  ms, 70 dB SPL pulses. Numbers correspond to time after presentation of pulse in seconds. Signal is averaged 2.5 pixels in x and y dimensions, and 2.5 frames through time. Image labelled ‘peak’ (right) corresponds to mean of 5 frames of peak fluorescence, averaged 5 pixels in x and y, only showing pixels with > 50% maximum ΔF/F. Dots represent weighted mid-point of fluorescence (see Materials and Methods). **e** Position of weighted mid-point of fluorescence plotted against frequency of pulses; data from each animal indicated by shade of grey (N = 3, at least 5 presentations per animal). Alexa-568 staining shown to scale for comparison. Scale bars: 100 μm

**Fig. 5:**
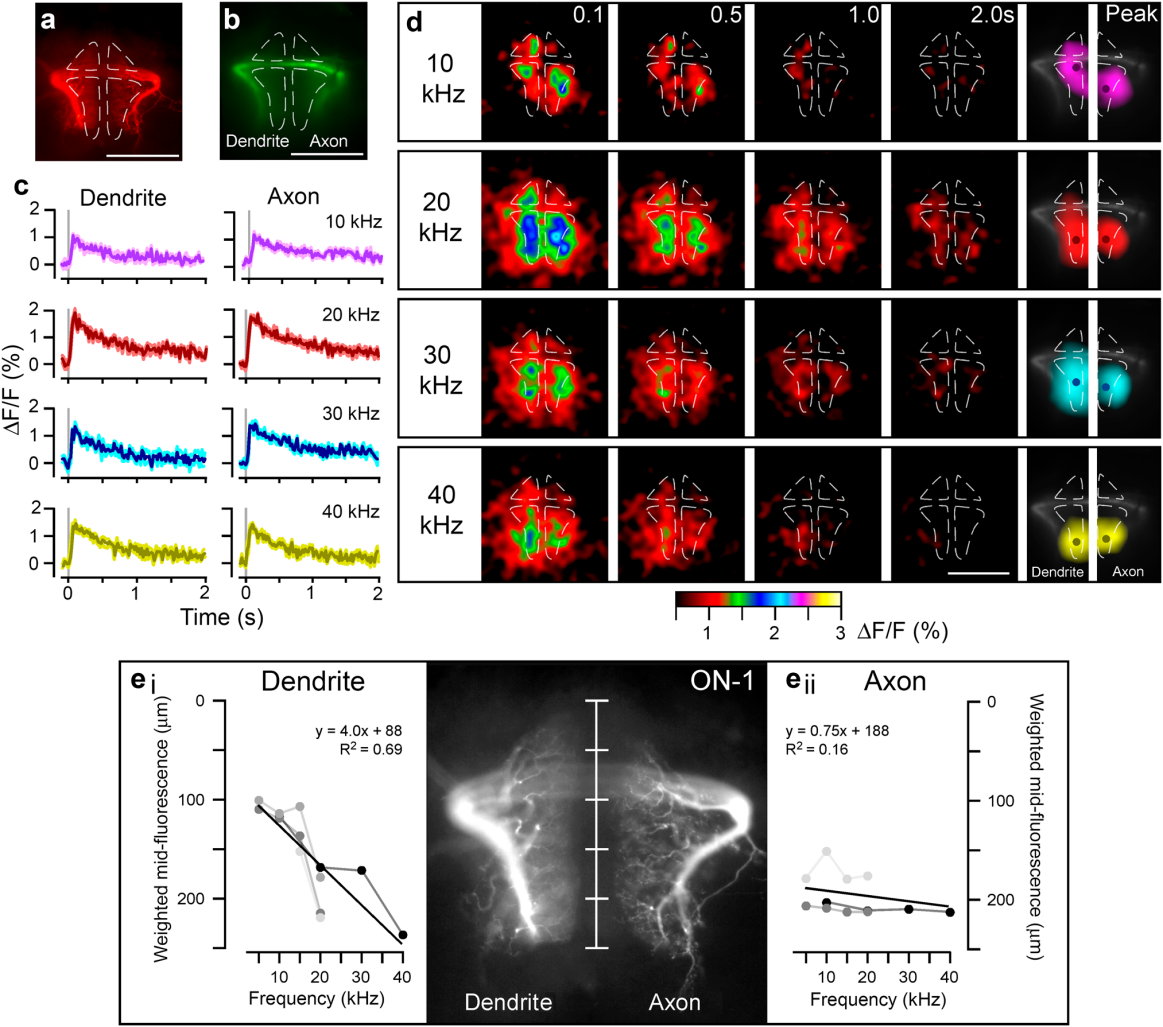
Ca^2+^ response of ON-1 to pure-tone sound pulses. **a** Single z-plane image from Alexa-568 staining after fixation and clearing, with ROIs indicated around dendrite and axon. **b** Baseline fluorescence of OGB-1 without stimulation in live preparation averaged from 100 frames prior to stimulation. **c** Mean ΔF/F in single animal for the dendritic field (left) and axonal arborisations (right) after presentation of 20 ms, 70 dB SPL sound pulses of 10, 20, 30 and 40 kHz indicated by grey bars, 5–10 presentations per frequency. **d** Mean Ca^2+^ fluorescence from 5–10 presentations of sound pulses. Numbers correspond to time after presentation of pulse in seconds. Signal is averaged 2.5 pixels in x and y dimensions, and 2.5 frames through time. Scale bar indicated in bottom row. Image labelled ‘peak’ (right) corresponds to mean of 5 frames of peak fluorescence, averaged 5 pixels in x and y, only showing pixels with > 50% maximum ΔF/F. Dots represent weighted mid-point of fluorescence. **e**i, ii Position of weighted mid-point of fluorescence plotted against frequency of pulses for the dendritic field and the axonal terminals; data from each animal indicated by shade of grey (N = 4, at least 5 presentations per animal). Alexa-568 staining shown to scale for comparison. Scale bars: 100 μm

TN-1 and ON-1 both receive excitatory inputs from the axonal terminals of the auditory afferents, which are arranged tonotopically; low frequency is represented in anterior regions and high frequency in posterior regions of the auditory neuropil (Oldfield 1983; Römer 1983; Stölting and Stumpner 1998; Baden and Hedwig 2010). Therefore, the possibility of localised Ca^2+^ signals in the dendrites of both TN-1 and ON-1 was examined (Figs. 4 and 5; Movie 3, 4).

To test the Ca^2+^ response in TN-1, 20 ms pulses of 10–40 kHz were presented in steps of 10 kHz. First, the amplitude of the fluorescence increase across the entire dendritic region was measured (Fig. 4a–c). A pulse of 10 kHz induced an increase of 0.4 ± 0.2%; 20 kHz, 1.5 ± 0.4%; 30 kHz, 0.8 ± 0.2%; and 40 kHz, 0.9 ± 0.1% (N = 3, 3–5 presentations per frequency). The Ca^2+^ increase started within the same frame as the onset of each sound pulse, and reached its peak intensity within 40–60 ms, or 2–3 frames of imaging. The decay of Ca^2+^ was much slower, occurring within 2 s (τ_d_ was not measured). When investigating the spatial distribution of the Ca^2+^ signals in the dendrites of TN-1, the highest ΔF/F in response to a 10 kHz pulse occurred in its anterior regions (Fig. 4d, top; Movie 3), to 20 and 30 kHz pulses, it occurred in a broad central region (Fig. 4d, middle), and to a 40 kHz pulse, it occurred in its posterior regions (Fig. 4d, bottom). To quantify the position of the Ca^2+^ increase, the weighted mid-point of the fluorescence distribution was calculated for the peak response to each pulse (Fig. 4d, right; see Materials and Methods); e.g. on presentation of a 10 kHz pulse, the mid-point of fluorescence occurred 118 ± 3 μm from the anterior tip of the dendritic field, whereas for 20 kHz stimulation, it occurred 152 ± 10 μm from the anterior tip. In accordance with this pattern, the antero-posterior position of the fluorescence mid-point was linearly correlated with the frequency of sound presented, with high frequency sound inducing responses more posteriorly at an increment of 1.9 μm per kHz (Fig. 4e, *R*^2^ = 0.82, *p* < 10^−4^).

### Tonotopy in the Ca^2+^ response of ON-1

The dendrites and the axonal arborisations of ON-1 were simultaneously imaged. As the auditory afferents only provide inputs to the dendrites, we expected that tonotopic Ca^2+^ signals should only occur along ON-1’s dendritic region, and not its axonal arborisations. To test for localised Ca^2+^ signals, 20 ms pulses were presented of either 5–20 kHz in 5 kHz steps (N = 3), or 10–40 kHz in 10 kHz steps (N = 1). First, the amplitude of fluorescence increase across the whole dendritic region was measured (Fig. 5a–c): a pulse of 5 kHz induced a mean Ca^2+^ increase of 1.4 ± 1.0% (N = 3); 10 kHz, 1.7 ± 0.9% (N = 4); 15 kHz, 2.2 ± 1.5% (N = 3); 20 kHz, 2.1 ± 1.3% (N = 4); 30 kHz, 1.4 ± 0.4% (N = 1); and 40 kHz, 1.4 ± 0.4% (N = 1). In the axonal arborisations a pulse of 5 kHz induced a mean ΔF/F of 1.4 ± 1.0% (N = 3); 10 kHz, 1.6 ± 0.8% (N = 4); 15 kHz, 1.8 ± 1.4% (N = 3); 20 kHz, 1.7 ± 0.9% (N = 4); 30 kHz, 1.5 ± 0.5% (N = 1); and 40 kHz, 1.4 ± 0.4% (N = 1). The response in both the dendritic and axonal arborisations started within the same frame as the onset of sound, and reached its peak intensity within 40–60 ms, or 2–3 frames of imaging. The decay was slower, occurring within 2 s (τ_d_ was not measured).

Similar to TN-1, the regions of the dendritic arborisations showing the highest ΔF/F differed depending on the sound frequency presented: a 10 kHz pulse induced a Ca^2+^ increase in anterior regions (Fig. 5d, top; Movie 4), whereas 20 and 30 kHz pulses induced a broadly distributed Ca^2+^ increase over central regions (Fig. 5d, middle), and a 40 kHz pulse induced a Ca^2+^ increase in posterior regions (Fig. 5d, bottom). The antero-posterior position of the midpoint of fluorescence was linearly correlated with the frequency of the presented pulse, with an increment of 4.0 μm to the posterior per kHz (Fig. 5e i, *R*^2^ = 0.69, *p* = 2.6 × 10 − 4). For example, the weighted fluorescence mid-point (Fig. 5d, right), upon stimulation with a 10 kHz pulse, fell 115 ± 3 μm from the anterior tip of the dendrites, whereas to a 20 kHz pulse, it fell 193 ± 16 μm from the anterior tip. Unlike in the dendrites, in the axonal arborisations, the region with the highest ΔF/F did not differ with the frequency of the pulse presented; a Ca^2+^ increase occurred in posterior regions in response to pulses of all frequencies tested (Fig. 5d). There was no correlation between the antero-posterior position of the fluorescence mid-point and the sound frequency of pulse presented (Fig. 5e ii, *R*^2^ = 0.16, *p* = 0.19). For example, the weighted mid-point of fluorescence (Fig. 5d, right), upon stimulation with a 10 kHz pulse, fell 193 ± 17 μm from the anterior tip of the axonal terminals, and upon stimulation with a 20 kHz pulse, fell 205 ± 15 μm from the anterior tip. Together, these results indicate that localised Ca^2+^ increases occur in the dendrites of both TN-1 and ON-1, where they follow the tonotopic arrangement of the auditory afferents. Tonotopic Ca^2+^ signals did not occur in the axonal arborisations of ON-1. Note that the fluorescence mid-points overall cover the same anterior–posterior range in TN-1 and ON-1, however with a different steepness of the fitted regression function.

### Comparison of spiking response and Ca^2+^ signals

**Fig. 6:**
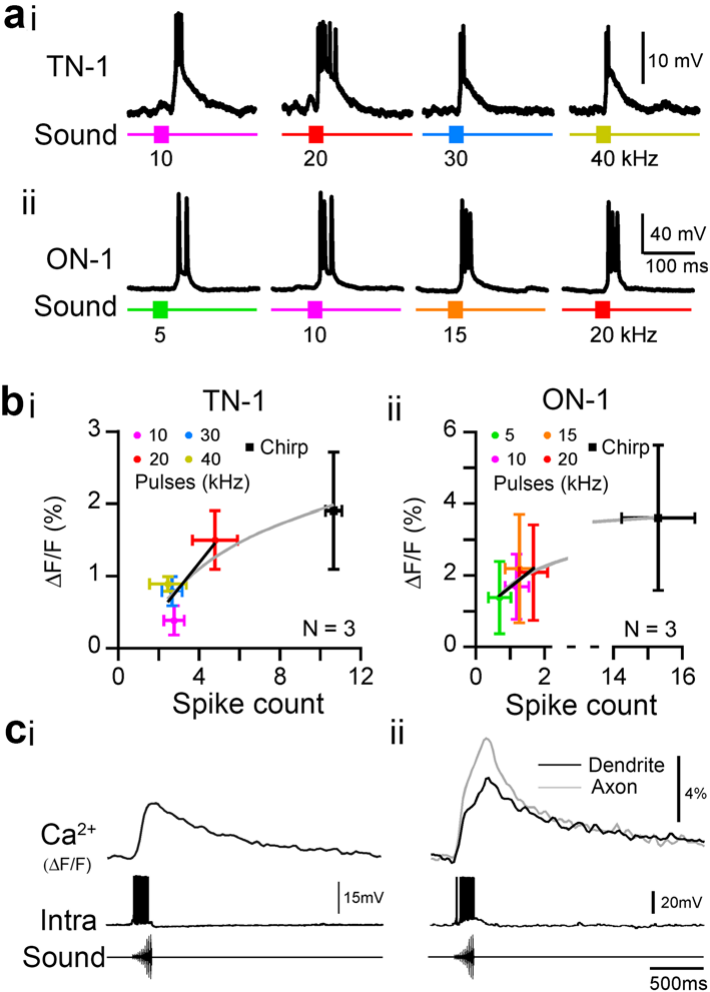
Comparison of Ca^2+^ signals and spiking responses. **a**i, ii Representative intracellular recording of TN-1 and ON-1 to 20 ms, 70 dB SPL pulses of different frequencies. **b**i, ii Mean ΔF/F (%) plotted against mean spike count in TN-1 and ON-1 to pulses of different frequencies, and species-specific chirp. Exponential fit through responses to pulses and chirp is shown (grey line). **c**i, ii Spiking response of TN-1, and ON-1 to a species-specific chirp. Raw Ca^2+^ signal shown above, in ΔF/F (%). Signal measured from ROIs in Fig. 4b for TN-1 and Fig. 5b for ON-1. Representative intracellular recordings and imaging data were not obtained simultaneously

For comparison with the Ca^2+^ signals the neurons’ spiking responses were recorded to the same stimuli used for imaging. In TN-1, a 20 ms pulse of 10 kHz frequency induced 2.8 ± 0.5 spikes; 20 kHz, 4.8 ± 1.1 spikes; 30 kHz, 2.7 ± 0.5 spikes; and 40 kHz, 2.5 ± 0.9 spikes (Fig. 6ai, N = 4). ON-1 generated a lower number of spikes, with 5 kHz stimulation inducing 0.8 ± 0.4 spikes; 10 kHz, 1.4 ± 0.4 spikes; 15 kHz, 1.5 ± 0.5 spikes; and 20 kHz, 2.0 ± 0.5 spikes (Fig. 6aii, N = 4). ON-1 responses to 30 and 40 kHz stimulation were not tested as imaging information was only available for one animal.

When also considering the response to a species-specific chirp, to which TN-1 produced 10.6 ± 0.4 spikes (Fig. 6ci), and ON-1 produced 15.0 ± 1.3 spikes (Fig. 6cii), both TN-1 and ON-1 showed a logarithmic increase in mean ΔF/F in their dendritic arborisations with the spike count: in TN-1, ΔF/F varied with 0.9 x ln(spike count) (Fig. 6bi, grey line, *R*^2^ = 0.8. N = 3); and in ON-1, it varied with 0.74 x ln(spike count), (Fig. 6bii, grey line, *R*^2^ = 0.96, N = 4). This suggests a saturating function, with ΔF/F reaching a plateau with higher spike counts at around 12–15 spikes per chirp. For both TN-1 and ON-1, the Ca^2+^ signal is aligned with the intracellular signal in response to a chirp for comparison (Fig. 6c).

### Contralateral inhibition in ON-1-afferent cold deactivation and neuronal response

**Fig. 7:**
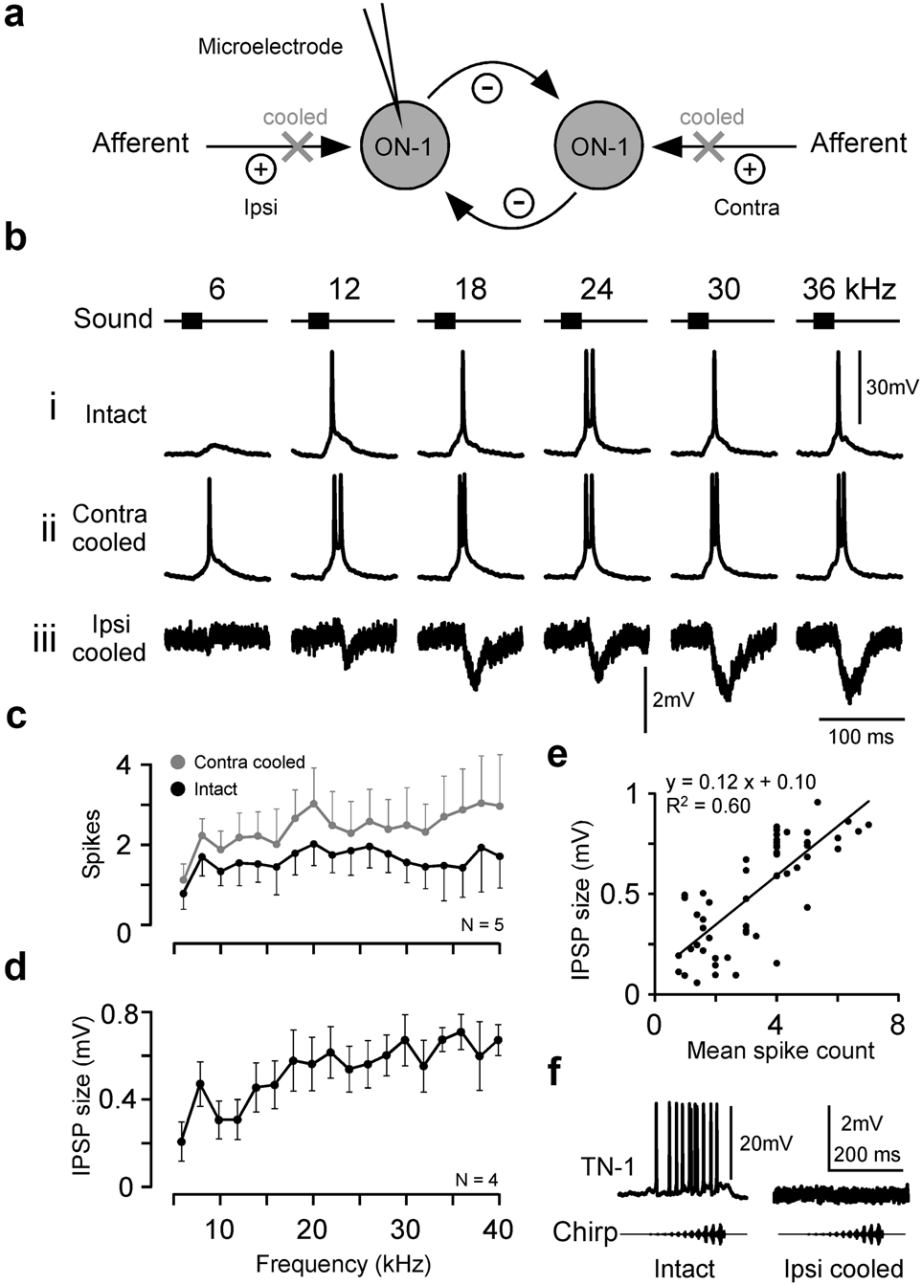
Effect of contralateral inhibition on the response of ON-1. **a** Diagram showing reciprocal inhibition between ON-1 neurons and the effect of afferent deactivation. Afferents are labelled ipsilateral (Ipsi), and contralateral (Contra) to the dendrites of the recorded ON-1. Afferents provide excitatory input to ON-1s (plus signs), which can be reversibly deactivated by cooling (crosses). Each ON-1 provides inhibition to the contralateral ON-1 (minus signs). **b** Spiking response of ON-1 to 10 ms pulses of different frequencies when: i intact; ii the contralateral auditory afferents deactivated, and iii the ipsilateral auditory afferents deactivated. **c** Mean spiking response of ON-1 in five animals to 6–40 kHz stimulation when intact (black circles) and when the contralateral afferents were deactivated (grey circles). **d** Mean frequency tuning of IPSP size while the ipsilateral afferents were deactivated (N = 4). **e** Pooled data from three animals showing correlation between spike number when contralateral afferents were deactivated, and IPSP size when ipsilateral afferents were deactivated. Points represent mean response to one frequency (at least 3 pulses per point). **f** Response of TN-1 to a chirp when intact (left), and when the ipsilateral afferents were deactivated (right). No hyperpolarisation was observed

In crickets the bilateral ON-1 neurons in the prothoracic ganglion reciprocally inhibit each other. In bush-crickets contralateral inhibition of the ON-1 neurons occurs and may be mediated by direct reciprocal inhibition and/or an indirect pathway. Reciprocal inhibition reduces the response to background stimulation and increases the contrast between the left and right ON-1 response (Kleindienst et al. 1981; Selverston et al. 1985). To assess the role of contralateral inhibition on processing in the *M. elongata* ON-1, the auditory afferents contralateral or ipsilateral to the dendrites and cell body of the recorded ON-1, were reversibly cooled, and so deactivated (Fig. 7a). We correspondingly refer to the afferents as ‘contralateral’ and ‘ipsilateral’.

In this paradigm the tuning of ON-1 before and during cooling of ipsilateral or contralateral afferents was compared within a wide frequency range, from 6 to 40 kHz in 2 kHz steps (Fig. 7b–e). Normally, ON-1 showed a fairly flat frequency tuning between 8 and 40 kHz, generating between 1 and 2 spikes per stimulus (Fig. 7bi, c). When the contralateral ear was cooled, the number of spikes increased to between 2 and 4 spikes, with a particularly increased response to frequencies over 20 kHz (Fig. 7bii, c, N = 5). When the ipsilateral side was cooled, spikes were abolished and the response was dominated by hyperpolarisation, indicative of compound IPSPs (Fig. 7biii), which had mean amplitudes of up to 2 mV and were largest at frequencies over 20 kHz (Fig. 7d, N = 4). This is in-line with ON-1 receiving a contralateral inhibition and suggests complete deactivation of the auditory afferents when cooling the tibia with the hearing organ. The spike count in ON-1 when deactivating the contralateral afferents was highly correlated with compound IPSP size when deactivating the ipsilateral afferents, with a mean IPSP size of 0.12 ± 0.01 mV/spike (Fig. 7e, *R*^2^ = 0.60, *p* = 7 × 10^−12^ pooled data from 3 animals), with each spike corresponding to 0.12 ± 0.01 mV of inhibition.

In TN-1, no IPSPs were observed when deactivating the ipsilateral afferents (Fig. 7f), indicating that the neuron does not receive a contralateral inhibition; therefore, the response of TN-1 was not further examined.

### Contralateral inhibition in ON-1—Ca^2+^ signals

**Fig. 8:**
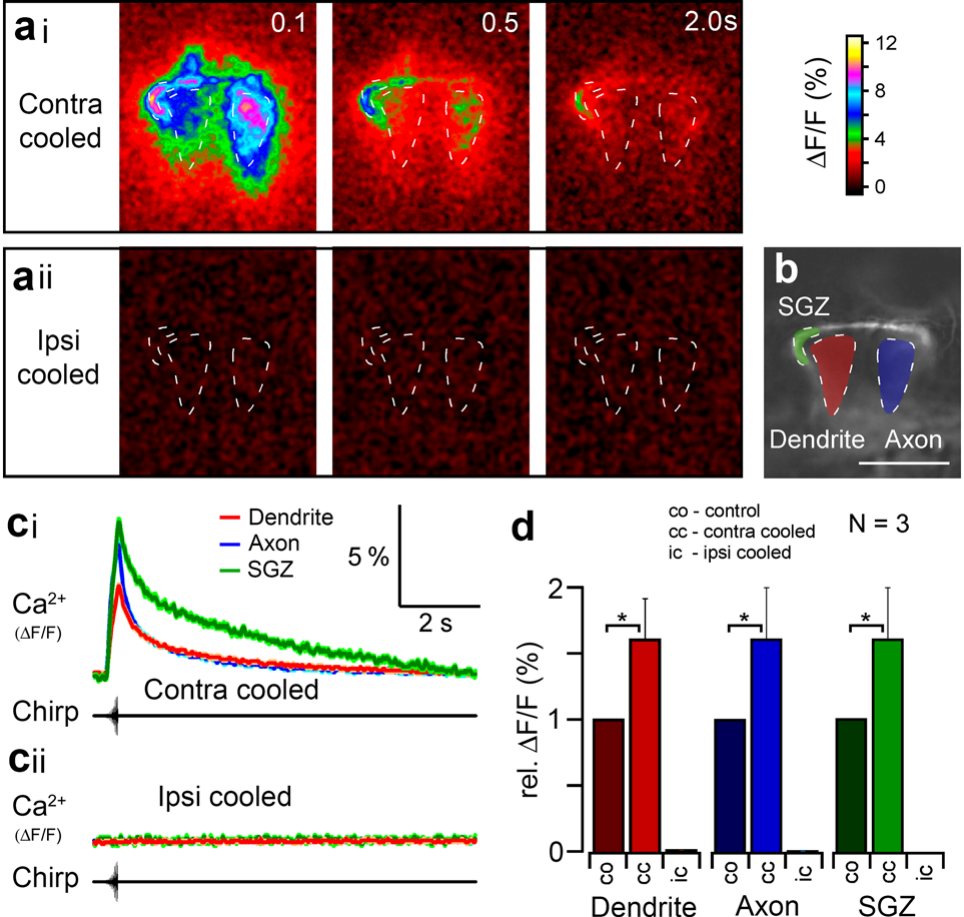
Effect of deactivating auditory afferents on Ca^2+^ fluorescence signal in ON-1. **a**i, ii Ca^2+^ related fluorescence signal after deactivating contralateral or ipsilateral afferents. **b** Dashed lines show ROIs drawn for dendrites (red), axonal terminals (blue) and SGZ (green). Baseline fluorescence of OGB-1 without stimulation from average of 100 frames before stimulation. **c**i, ii Mean fluorescence (ΔF/F %) in ROIs on presentation of chirps. Average from 19 chirps during cold deactivation of contralateral or ipsilateral afferents. **d** Mean relative ΔF/F in each ROI in control (CO) and after cold deactivation of contralateral (CC) or ipsilateral (IC) auditory afferents (N = 3). Asterisks represent significant difference from control. *p < 0.0025, ***p < 0.0005: paired t-tests. Scale bar: 100 μm

To test the influence of contralateral inhibition on the Ca^2+^ response of ON-1, and to identify any. contralaterally derived Ca^2+^ signals, ON-1 was imaged before and during deactivation of the contralateral or ipsilateral afferents (Fig. 8). When the contralateral afferents were deactivated, a large fluorescence increase occurred in ON-1 in response to a chirp (Fig. 8ai, ci, d; N = 3). Compared with the normal response ΔF/F was greater in all regions of the neuron, with the axon showing 1.6 ± 0.3 times greater ΔF/F, and both the dendrite and SGZ showing 1.6 ± 0.4 times greater ΔF/F (Fig. 8d; *p* = 0.05, 0.04, 0.05, respectively, paired t-tests, N = 3). The increased ΔF/F is likely due to the stronger depolarisation and higher number of spikes when contralateral inhibition was blocked.

When the ipsilateral afferents were deactivated, no fluorescence increase was discernible in response to a chirp in any region of the neuron (Fig. 8aii, cii, d); peak ΔF/F in the dendrite was reduced to 0.07 ± 0.01%; in the axon, to 0.05 ± 0.01%; and in the SGZ, to 0.02 ± 0.01% (Fig. 8d). This implies that all Ca^2+^ increases to acoustic stimulation are due to ipsilateral excitatory inputs to the dendrites of ON-1 and that contralateral inhibitory inputs do not induce a Ca^2+^ change.

### Forward masking in ON-1

**Fig. 9:**
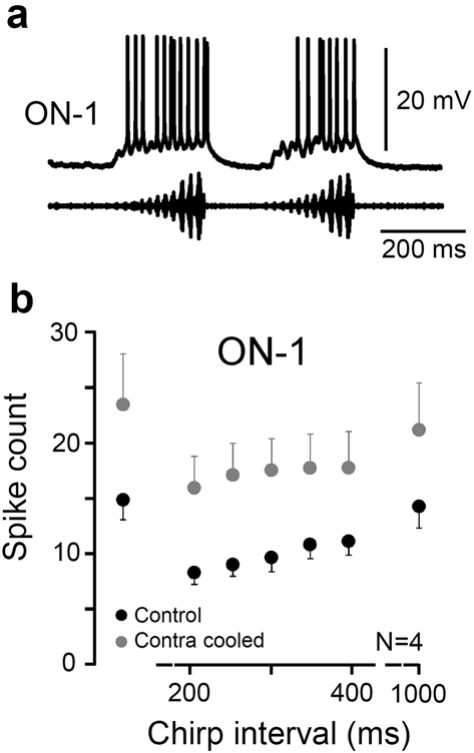
Adaptation to repetitive presentation of chirps. **a** Intracellular recording of ON-1 when two chirps were presented with a 350 ms inter-chirp interval. **b** Spikes per chirp for various inter-chirp intervals for ON-1 (N = 4) with at least three presentations per animal. The spike count before (black circles) and after cold deactivation (grey circles) of the contralateral auditory afferents is shown

In the auditory interneurons of bush-crickets (Triblehorn and Schul 2013; Presern et al. 2015), and crickets (Sobel and Tank 1994; Baden and Hedwig 2007), acoustically induced Ca^2+^ increases drive the activation of Ca^2+^-sensitive K^+^ channels to cause a short-term reduction in spiking. This provides forward masking; a period of time after a stimulus in which the response to a subsequent stimulus is reduced. To test for forward masking in ON-1, pairs of chirps were presented at intervals of 200–1000 ms (350 ms, Fig. 9a).

To test for any effect of contralateral inhibition on forward masking, the response to a chirp after 200 ms interval was compared in a control condition and when the contralateral afferents were cold deactivated. In the control condition the spike count to the first chirp was 14.9 ± 1 reducing to 8.3 ± 1.1 spikes to the second chirp (p = 0.01, paired t-tests, N = 4). When the contralateral afferents were cold deactivated, the number of spikes to the first chirp increased to 23.4 ± 4.5 spikes, reducing to 15.8 ± 2.7 spikes in response to the second chirp at 200 ms interval (Fig. 9b, *p* = 0.05, paired t-test, N = 4). In both cases the drop in spike count gradually reduced with increasing chirp intervals (Fig. 9b). This indicates that in ON-1 ipsilateral mechanisms contribute to forward masking which is in line with the activation of Ca^2+^ -sensitive K^+^ channels. Besides this, adaptation in the auditory afferents may also have an impact (Ocker and Hedwig 1993; Nabatiyan et al. 2003), although this was not tested.

### Frequency-specific adaptation

Given the tonotopically organised Ca^2+^ increases (Figs. 4 and 5), any calcium derived adaptation effects in the dendrites should only occur in a restricted region. We therefore tested for frequency-specific adaptation in TN-1 and ON-1. To distinguish adaptation in TN-1 and ON-1 from adaptation in the auditory afferents intracellular recordings were also made from single afferents (not shown). Across all frequencies tested (10, 20, 30, and 40 kHz) a similar degree of adaptation occurred with the spike count reduced to 72 ± 15% of the initial response after 1 s stimulation with a pure-tone stimulus at 70 dB SPL (N = 3, 5 presentations per animal).

### TN-1

**Fig. 10:**
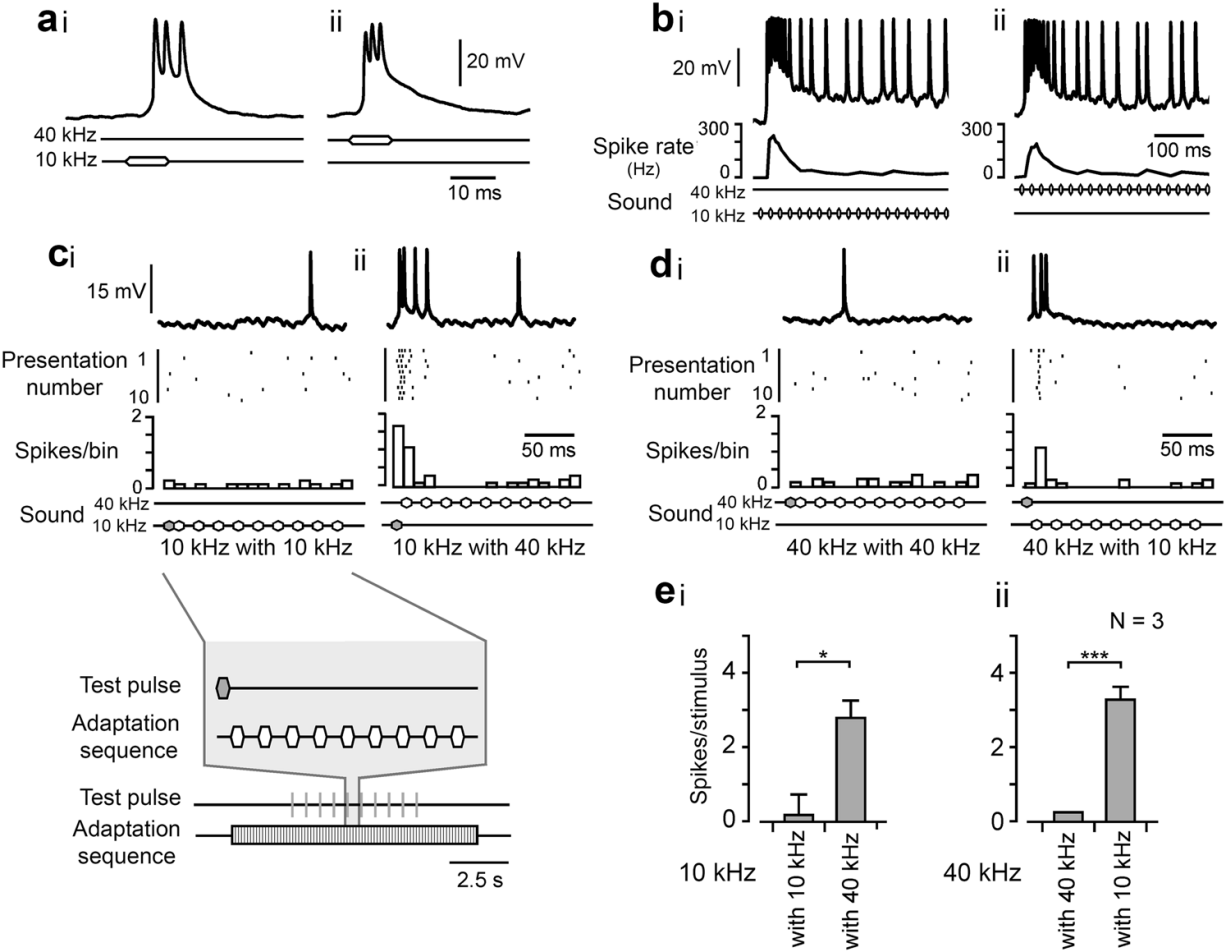
Frequency-specific adaptation in TN-1. Sound stimulation is represented for all traces: top line indicates 40 kHz stimulation; bottom line represents 10 kHz stimulation. **a** Response of TN-1 to a single 10 ms 70 dB SPL test pulse of 10 kHz (**i**) and 40 kHz (ii). **b**i, ii Response to series of 10 ms pulses of 10 and 40 kHz, presented at 20 ms pulse period. Instantaneous spike rate plots are shown below. **c**, **d** Frequency-specific adaptation in TN-1, inset of (**c**) gives stimulus paradigm. **c**i, ii Response to a test pulse of 10 kHz, after adaptation to a series of 10 kHz pulses, and after adaptation to a series of 40 kHz pulses. Top trace: intracellular recording. Middle: occurrence of spikes over trials. Bottom trace: frequency plot of the spike response. **d**i, ii Response to a test pulse of 40 kHz after adaptation to a series of 40 kHz pulses and after adaptation to a series of 10 kHz pulses. **e**i Mean spike count for a 10 kHz test pulse presented with a series of 10 kHz pulses and with a series of 40 kHz pulses. **e**ii Mean spike count for a 40 kHz test pulse presented with a series of 40 kHz pulses and with a series of 10 kHz pulses. Asterisks represent significant difference of responses. *p < 0.05, ***p < 0.001; unpaired t-tests

 Before testing for frequency specificity of adaptation in TN-1, its response to isolated tone pulses was measured. An isolated pulse of 70 dB SPL and 10 ms duration gave 4.2 ± 0.6 spikes at 10 kHz and at 40 kHz it gave 2.7 ± 0.4 spikes (Fig. 10a). The inter-dependence of adaptation to pulses of 10 and 40 kHz was then tested. First, a series of pulses (10 ms duration, 10 ms interpulse interval (i.e. 20 ms pulse period) was presented to drive adaptation in the neuron at either 10 or 40 kHz (Fig. 10b). After 2.5 s we then presented 10 kHz test pulses during the 40 kHz adaptation series, and we presented 40 kHz test pulses during the 10 kHz series (Fig. 10c, d). These were given in the silent intervals between the pulses of the adaptation series and repeated at 200 ms intervals (Fig. 10ci, bottom). The number of spikes in response to the 10 or 40 kHz test pulses was measured and compared. When the frequency of the test pulses was different to the adaptation frequency, the response of TN-1 to the test pulses was significantly different. A 10 kHz test pulse combined with a series of 10 kHz pulses induced 0.2 ± 0.6 spikes, i.e. 4.7% of the initial response (Fig. 10ci, ei) and combined with a series of 40 kHz pulses it induced 2.8 ± 0.5 spikes (Fig. 10cii, ei) giving a significantly different response (*p* = 0.029, unpaired t-test, N=3). A 40 kHz test pulse combined with a series of 40 kHz pulses induced 0.3 ± 0.0 spikes, i.e. 11.1% of the initial response (Fig. 10di, eii), and combined with a series of 10 kHz pulses it induced 3.3 ± 0.3 spikes (Fig. 10dii, eii) and showed a significant difference (*p *= 0.6 x10^−4^, unpaired t-test, N=3). This suggests that frequency-specific adaptation does occur in TN-1, i.e., adaptation to a 10 kHz is independent of processing 40 kHz signals and adaptation to 40 kHz signals is independent of processing 10 kHz signals. Overall the adaptation in TN1 was an order of magnitude greater than in the afferents.

### ON-1

**Fig. 11:**
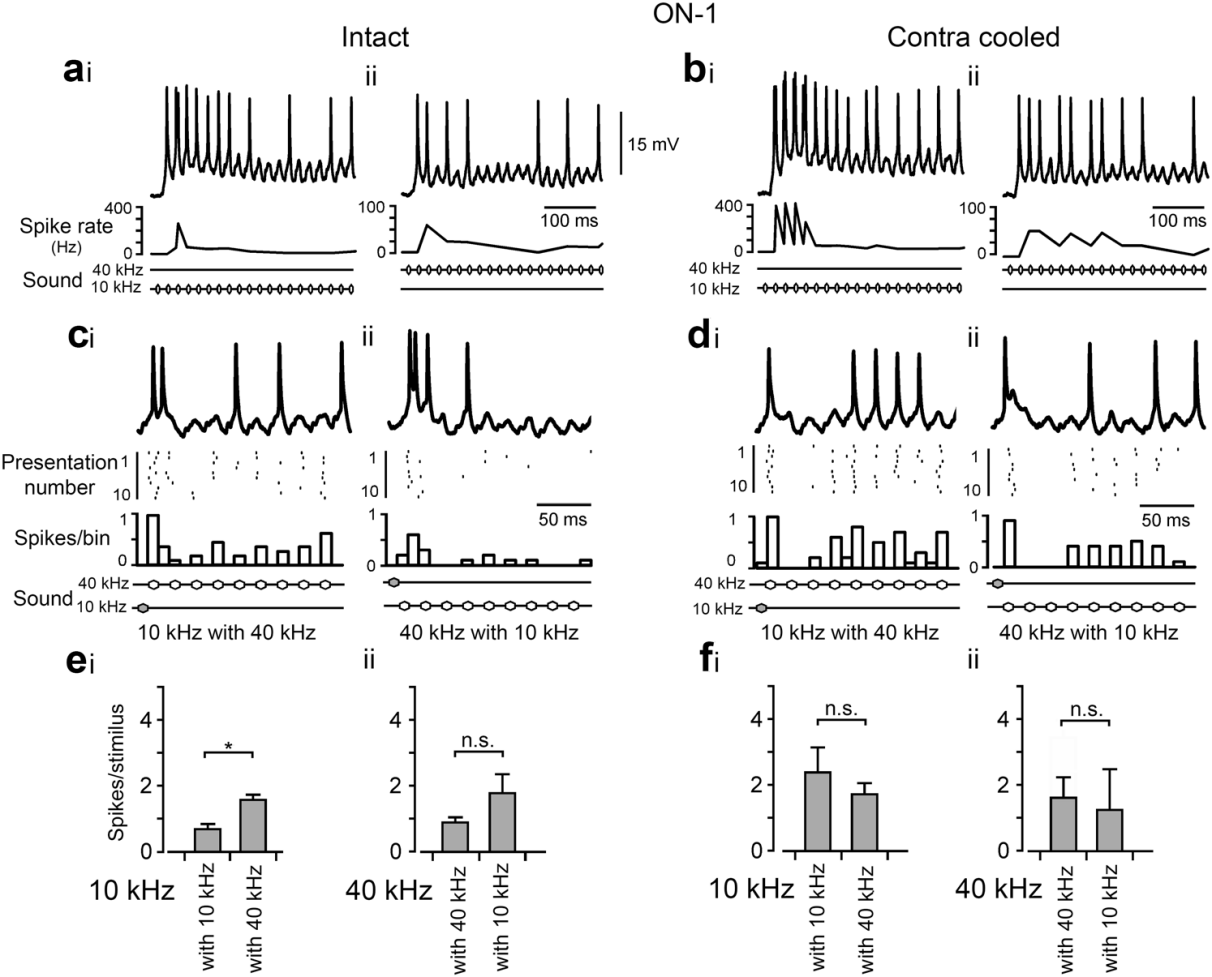
Frequency specific adaptation in ON-1, and the impact of contralateral inhibition on adaptation. **a** Response of ON-1 to a series of 10 ms, 70 dB SPL sound pulses at 20 ms pulse period for pulses of 10 kHz (i) and 40 kHz (ii). Instantaneous spike rate plots are shown below; note the different scales for 10 and 40 kHz stimulation. **b** As (**a**) but with contralateral afferents deactivated. **c**i Response to a test pulse of 10 kHz frequency, after adaptation to series of 40 kHz pulses; **c**ii Response to test pulse of 40 kHz after adaptation to a series of 10 kHz pulses. Stimulus paradigm as in Fig. 10c. Top trace: intracellular recording. Middle: occurrence of spikes over trials. Bottom: frequency plot of the spike response. **d** As for (**c**) but with contralateral afferents deactivated. **ei** Mean spike count for a 10 kHz test pulse after adaptation to a 10 kHz and after a 40 kHz adaptation series. **e**ii Response to a 40 kHz test pulse correspondingly. **f** As for (**e**) but with contralateral afferents deactivated. In (**i**) values are given for 10 kHz test pulse presented with a series of 10 kHz pulses or a series of 40 kHz pulses. In (ii) data are for 40 kHz test pulses presented with a series of 40 kHz pulses or with a series of 10 kHz pulses. Asterisk represents significant difference between responses *p < 0.05, unpaired t-tests

 Before adaptation, ON-1 generated an initial response of 2.0 ± 0.0 spikes to a 10 kHz pulse (Fig. 11ai, N = 5), and 2.2 ± 0.7 spikes to a 40 kHz pulse (Fig. 11aii, N = 5). To measure adaptation, a 2.5 s series of pulses was presented at the same frequency. At the end of a series of 10 kHz pulses the response adapted to 0.7 ± 0.2 spikes/pulse, i.e. 35% of the initial response (Fig. 11ai, ei), and at the end of a series of 40 kHz pulses the response adapted to 0.9 ± 0.2 spikes/pulse, i.e. 40.9% of the initial response (Fig. 11aii, eii). When testing for frequency-specific adaptation a 10 kHz pulse was presented after adaptation to a series of 40 kHz pulses, which generated 1.6 ± 0.2 spikes (Fig. 11ci, ei), and was significantly different to the response in combination with 10 kHz pulses (*p* = 0.013, unpaired t-test, N = 5). A 40 kHz pulse presented after a series of 10 kHz pulses generated 1.8 ± 0.6 spikes (Fig. 11cii, eii), which was not different to the response in combination with 40 kHz pulses (*p* = 0.385, unpaired t-test, N = 5). Thus, compared with TN-1, frequency-specific adaptation in ON-1 was less pronounced, and occurred only to a similar degree that in the afferents.

Considering the effect of contralateral inhibition in ON-1, evidence for frequency-specific adaptation was tested during cold deactivating the contralateral afferents. With contralateral inhibition abolished, the response to stimuli presented alone significantly increased to 4.2 ± 0.5 spikes for a 10 kHz pulse (Fig. 11bi), and to 3.4 ± 0.6 spikes for a 40 kHz pulse (Fig. 11bii, *p* = 0.01, *p* = 0.03, respectively, paired t-tests, N = 5).

Adaptation still occurred, with the response after a 2.5 s series of 10 kHz pulses adapting to 2.4 ± 0.7 spikes/pulse, (Fig. 11bi, fi, N = 5), i.e. 57% of the initial response. The response to 40 kHz stimulation adapted to 1.6 ± 0.6 spikes/pulse (Fig. 11bii, fii, N = 5), i.e. 47% of the initial response. This shows that when contralateral inhibition is removed, there is an over-all increase in ON-1 spike activity, but adaptation still occurs, indicating that ipsilateral mechanisms contribute significantly to adaptation. With the contralateral afferents deactivated, a 10 kHz pulse combined with a series of 40 kHz pulses generated 1.7 ± 0.3 (Fig. 11di, fi, N = 5), which was not significantly different to the response in a series of 10 kHz pulses (*p* = 0.39, unpaired t-test, N = 5). A 40 kHz pulse over a series of 10 kHz pulses generated 1.3 ± 1.2 spikes (Fig. 11dii, fii, N = 5), also this response was not significantly different to the response with a series of 40 kHz pulses (*p* = 0.83, unpaired t-test, N = 5). Thus, after removal of the contralateral inhibition frequency-specific adaptation was not evident in ON-1.

## Discussion

We combined intracellular recordings and optical imaging to study processing of neural activity in identified auditory interneurons of a bush cricket, after loading the neurons with a calcium sensitive indicator. The large size of the *Mecopoda* interneurons allow the study of the integration of sensory information across the entire dendritic and axonal arborisations.

### Modes of Ca^2+^ signals

After dye loading, the neurons revealed clear fluorescence responses to acoustic stimuli. The timescales of Ca^2+^ signals observed are likely to be influenced by the presence of the calcium indicator, which acts as a chelator of Ca^2+^, slowing down its removal from the cytosol (Helmchen et al. 1997; Sabatini et al. 2002; Augustine et al. 2003). To minimise this effect and to obtain an even distribution throughout the neuron, the dye was used in low concentrations and allowed to spread for 30–60 min. Imaging of fluorescence changes in the dendrites during acoustic stimulation revealed an increase in the fluorescence signal which reached its maximum within 330 ms followed by a long decay over a time of 1–2 s. These changes were like those seen in the cricket auditory pathway when low-affinity indicators were used, to capture the temporal dynamics of the Ca^2+^ signals more closely (Baden and Hedwig 2007, 2010). This suggests that the indicator effects on the timing and especially the spatial pattern of Ca^2+^ signals were minor.

In the dendrites of both TN-1 and ON-1, Ca^2+^ influx is likely to be mediated by ligand-gated channels. Crickets have cholinergic auditory afferents (Stout et al. 1998), and bush-crickets likely as well. Nicotinic acetylcholine receptors allow the entry of Ca^2+^ (Decker and Dani 1990; Wegener et al. 2004), so synaptic transmission between auditory afferents and interneurons should cause a Ca^2+^ influx into the dendrites. A local excitatory post-synaptic potential will decay passively within a cell’s dendrites, and only a restricted region will be influenced by this potential (Cuntz et al. 2003). However this will be complicated when spikes passively back-propagate into the neurites and the cell body (Gwilliam and Burrows 1980). Actively invading spikes would impact the whole dendritic field and should mask any tonotopic responses; similar to what is observed in the axonal arborisations of ON-1 (Fig. 8). As for the dendrites of TN-1 or ON-1 (Figs. 4d and 5d) a masking of the tonotopic organisation by spiking activity was not observed, suggesting that spike derived membrane potential changes do not act alone to drive the Ca^2+^ increase in their dendrites. In-line with this, Ca^2+^ increases may be mediated by the concurrence of a neurotransmitter and membrane depolarisation, as reported in vertebrates (Nakamura et al. 1999). A separation of synaptic and spike mediated calcium responses can be achieved by a combination of intracellular recordings and Ca^2+^ imaging: for example, in the dendrites of cricket cercal giant interneurons there is no increase of the Ca^2+^ signal upon stimulation leading to subthreshold EPSPs while a Ca^2+^ signal occurs when single spikes are evoked (Ogawa and Mitani 2015). These giant neurons however likely have multiple spike initiating zones which may occur in their dendritic field, making a separation of dendritic and spiking processes more complex.

Upon blocking the ipsilateral excitatory inputs in experiments with ON-1, the contralateral inhibition, which acts on the dendrites, did not lead to a change in the calcium response, indicating that inhibitory inputs are not reflected in the fluorescence signal. A decrease in the fluorescence signal of ON-1 during contralateral inhibition as reported in a field cricket (Baden and Hedwig 2007) was rather mediated by a simultaneous reduced spiking response.

For the local ON-1 the Ca^2+^ signal could be compared between regions of the cell. As previously observed in the cricket ON-1 (Baden and Hedwig 2007), the highest magnitude and slowest decrease of Ca^2+^ signals always occurred in the SGZ. The increased signal in the SGZ and also the axon is likely due to voltage-gated Ca^2+^ channels, or a voltage-mediated release of Ca^2+^ from internal stores (Ryglewski et al. 2007). The SGZ does not overlap with afferents, so likely has no synaptic contacts with them, suggesting no ligand gated channels will be present in this region. The reason for the longer decay time in the SGZ is not clear, but it may be due to the higher volume and/or lower surface area in this region, which could both contribute to slower removal of Ca^2+^ (De Schutter and Smolen 1999). The presence of Ca^2+^ in the SGZ suggests that it may have a dampening effect on spike initiation if it activates Ca^2+^-sensitive K^+^ channels.

The Ca^2+^ signal in the axonal arborisations (Fig. 5) is likely driven by spike driven voltage-gated channels or a release from internal stores. This is corroborated by the lack of a Ca^2+^ increase in the axonal arborisations when the ipsilateral afferents were silenced, and spiking prevented (Fig. 8aii). Overall, a detailed picture can only be obtained when the Ca^2+^-conductances over the neuronal compartments are precisely mapped.

### Possible effects of Ca^2+^

Ca^2+^ is known to impact many processes within cells, from depolarisation and Ca^2+^ spikes to metabolism, microtubule dynamics, and gene expression (review: Branco and Häusser 2010). This means that Ca^2+^ could have a wide range of long-term and short-term effects in the neurons. Cytosolic Ca^2+^ has a crucial influence on a neuron’s function (review: Konieczny et al. 2012) it enters the cytosol through ligand or voltage-gated channels or from internal stores.

Ca^2+^ entry during neuron depolarisation can have a wide variety of impacts. Ca^2+^-dependent hyperpolarisation has been observed in several studies; as Ca^2+^ accumulates and activates Ca^2+^-sensitive K^+^ channels, it causes hyperpolarisation, a decrease in membrane resistance, and contributes to adaptation of the neuron (Wessel et al. 1999; Savić et al. 2001). This mode of action has previously been implicated in ON-1 of crickets (Baden and Hedwig 2007; Pollack 1988; Sobel and Tank 1994), and TN-1 of the bush-cricket *N. triops* (Triblehorn and Schul 2013; Presern et al. 2015) and attributed to selective attention in the auditory pathway (Pollack 1988). Likewise the frequency-specific adaptation in this study (Figs. 10 and 11) may be induced through the activation of Ca^2+^ sensitive potassium channels linked to localised Ca^2+^ increases in the dendrites. Such a mode of activation is also suggested for some cricket cercal giant interneurons, where stimulus specific adaptation to wind stimuli from specific directions appears to be linked to local dendritic Ca^2+^ increases (Ogawa and Oka 2015).

### Ca^2+^ signals mirror the tonotopic organisation of the auditory neuropil

TN-1 and ON-1 showed a spatially restricted Ca^2+^ increase in their dendritic field when pure-tone pulses were presented, such that low-frequency pulses (< 10 kHz) elicited a response in anterior regions, pulses of 20–30 kHz activated the middle area and high-frequency pulses (40 kHz) elicited a response in posterior regions (Figs. 4 and 5; Movie 3, 4). The anterior-to-posterior arrangement of low-to-high frequency sound reflects the tonotopic organisation of the axonal terminals of auditory afferents in the auditory neuropil. Moreover, the spatial dimensions of the Ca^2+^ responses match the spatial dimensions of the afferent axonal arborisations. Low- frequency and high-frequency afferents occupy a more restricted range in the anterior and posterior neuropil, respectively, while afferents responding to the midrange of frequencies, show a wider arborisation in the centre of the neuropil (Oldfield 1983; Römer 1983; Stölting and Stumpner 1998; Baden and Hedwig 2010). In contrast at the axonal arborisations of ON-1 the spatial distribution of the Ca^2+^ signal for different frequencies was uniform, like in the field cricket (Baden and Hedwig 2007). Therefore any reciprocal inhibitory inputs to the ON-1 neurons should be uniform across frequencies.

Localised Ca^2+^ signals have been observed in auditory neurons in another species of bush-cricket (Presern et al. 2015), and a cricket (Baden and Hedwig 2007). They have also been reported for insect neurons which receive inputs from maps of different modalities, including neurons responding to antennal stimulation (Bayley and Hedwig 2018) or wind stimulation of the cerci in crickets (Ogawa et al. 2008; Ogawa and Oka 2015) and to visual stimulation of the eyes in flies (Borst and Egelhaaf 1992; Spalthoff et al. 2010) and locusts (Peron et al. 2009). Local topographically organised Ca^2+^ signals have also been reported in neurons of vertebrates, including Purkinje fibres in the cerebellum, which overlap with a somatosensory map (Kitamura and Häusser 2011); CA1 pyramidal neurons in the hippocampus, which overlap with a spatial map (Sheffield and Dombeck 2014); and layer 2/3 pyramidal neurons in the cortex which overlap with a visual map (Jia et al. 2010) pointing to the relevance of spatial organisation of synapses underlying local dendritic processing as a common principle in nervous systems.

### The impact of contralateral inhibition

In crickets reciprocal inhibition between the ON-1 neurons on the left and right side of the prothoracic ganglion has been suggested to enhance directional sensitivity (Kleindienst et al. 1981; Selverston et al. 1985). Inhibition in bush-cricket ON-1 neurons has been reported for *Tettigonia viridissima* (Schul 1997) and contralateral inhibition has been implicated as a gain control mechanism (Römer and Krusch 2000). We tested the role of contralateral inhibition in activity-dependent response reduction for both forward masking after a species-specific chirp (Fig. 9), and adaptation to a rapidly repeated series of pure-tone pulses (Fig. 11). A higher spike count and Ca^2+^ signal (Fig. 8) was observed when contralateral inhibition was removed. However, activity-dependent response reduction still occurred, both during forward masking (Fig. 9), and adaptation to a series of pure-tone stimuli (Fig. 11). This suggests that activity-dependent response reduction is not the result of binaural mechanisms, but rather due to intrinsic Ca^2+^ dynamics, as reported for modelling ON-1 activity (Ponnath and Farris 2010).

A contralateral mediated response reduction linked to inhibition had been suggested in TN-1 of *Gampsocleis burger* (Suga and Katsuki 1961), but removing the contralateral afferent activity had no effect on TN-1 response in *N. retusus* (Schul et al. 2012), and our intracellular recordings also provide no supporting evidence for a contralateral mediated inhibition of TN-1 (Fig. 7f).

### Forward masking to chirps

When the bush crickets were stimulated with two chirps in rapid succession, the second resulted in fewer spikes in ON-1 (Fig. 9). The effect on spiking to the second chirp lasted around 1 s. In *M. elongata* species-specific chirp repetition rate is around 2 s (Hartbauer et al. 2005), which could suggest that males produce chirps at a rate to avoid forward masking and habituation in the female auditory system. Forward masking may be shaped by the activation of K^+^ channels by Ca^2+^, as both TN-1 and ON-1 produced a Ca^2+^ increase in response to a species-specific chirp, with a time-constant of decay of around 1–2 s (Figs. 2 and 3; Movie 1, 2). Forward masking is also important in the context of chorusing. Male *M. elongata* temporally couple their songs to those of other males, with one male calling slightly before another (Nityananda and Balakrishnan 2008; (Hartbauer et al. 2012b).

Female bush-crickets preferentially approach the male whose call is leading in a chorus (Fertschai et al. 2007). Forward masking is likely to influence this preference, as the response to the lagging male will be reduced. In ON-1, the bias to approach a leading male has previously been attributed to contralateral inhibition, but only with short inter-chirp intervals (< 300 ms), and only when chirps were directed to each ear independently (Römer et al. 2002). The work here suggests that, in ON-1, two mechanisms may work in tandem to reduce the response to a lagging male in a chorus: ipsilaterally derived forward masking, and contralateral inhibition.

### Frequency-specific adaptation

The spatial organisation of the dendritic Ca^2+^ responses imply specific processing of different sound frequencies, in a similar way as wind direction is matched onto the Ca^2+^ responses of dendritic fields in cricket cercal giant interneurons (Ogawa and Oka 2015). Our data indicate that frequency-specific adaptation occurs in TN-1 (Fig. 10); after adaptation to one frequency (either 10 or 40 kHz), a test pulse elicits a greater number of spikes when played with repetitive pulses of a different frequency (i.e. 10 kHz with 40 kHz, or 40 kHz with 10 kHz), than when played with repetitive pulses of the same frequency (i.e. 10 kHz with 10 kHz, 40 kHz with 40 kHz). To distinguish this from adaptation in the auditory afferents, their degree of adaptation was tested. We observed a drop to 72 ± 15% of the initial spike rate after 1 s stimulation with a pure-tone stimulus at 70 dB SPL. In TN-1, adaptation drove a reduction to around 7.9% of the initial response, where in ON-1, the reduction was to around 38% of the initial response. Adaptation in TN-1 is therefore an order of magnitude greater than that of the auditory afferents, the same cannot be said about ON-1. Also, in *N. retusus* frequency-specific adaptation in TN-1 is not due to the adaptation of the auditory afferents (Schul et al. 2012).

Frequency-specific adaptation has been previously described in TN-1 of the bush-cricket, *N. retusus* (Schul and Sheridan 2006; Schul et al. 2012) and has been implicated in the neuron’s ability to respond to bat calls at 30–50 kHz, while exposed to the species’ calling song, with a highest intensity around 15 kHz (Schul and Sheridan 2006; Schul et al. 2012). The calling song of *M. elongata*, has a broad frequency spectrum (Siegert et al. 2013), therefore the separation of calling song and bat calls in the frequency domain is less pronounced. Frequency-specific adaptation, however, could be important for the insects’ ability to detect species-specific chirps over background noise ((Hartbauer et al. 2012a). In chirping *M. elongata* TN-1 and ON-1 adapt to the rapidly repeated trills of one sub-species, whilst remaining able to respond to sparsely repeated chirps of their own presented simultaneously (Siegert et al. 2013). If a characteristic 2 kHz peak in the chirps of the species is removed, the neurons no longer respond, suggesting that frequency-specific adaptation to the signal of the trilling sub-species could contribute the ability of the chirping species to detect its own song in a situation exposed to masking noise by the trilling sub-species. These processes point to a possible role of the recently described prothoracic auditory DUM neurons of bush-crickets (Lefebvre et al. 2018), which project bilaterally into the auditory neuropil and function as a frequency filter bank.

## Conclusion

The experiments provide a deeper insight into the tonotopic organisation and processing of auditory inputs to the dendrites of the bush-cricket auditory interneurons TN-1 and ON-1 by Ca^2+^ imaging. In both neurons frequency specific adaptation likely occurs at the level of dendritic processing. Contralateral inhibition shapes the Ca^2+^ and spiking responses of ON-1.

### Supplementary Information

Below is the link to the electronic supplementary material.

Supplementary file1 (MP4 984 KB)

Supplementary file2 (MP4 1886 KB)

Supplementary file3 (MP4 2086 KB)

Supplementary file4 (MP4 1665 KB)
